# *Microorganisms* Special Issue “How Do Food and Probiotics Influence the Composition and Activity of the Gut Microbiota?”

**DOI:** 10.3390/microorganisms10112097

**Published:** 2022-10-22

**Authors:** Gisèle LaPointe, Michael A. Rogers

**Affiliations:** 1 Canadian Research Institute for Food Safety, University of Guelph, 43 McGilvray St., Guelph, ON N1G 2W1, Canada; 2 Department of Food Science, University of Guelph, 50 Stone Rd. East, Guelph, ON N1G 2W1, Canada

We are a product of the foods we chronically consume, and life expectancy correlates with the quality of our diet. What we eat influences the immune and cardiovascular systems, gut permeability and microbial dysbiosis, mental activity and health, and has long-term effects on fat storage. With rapid transformative advances to contemporary tools, how we study microbial ecosystems alters our perspective in both food and the gut. Sequencing technologies originally evolved from single gene profiles to complete genomic panoramas of microbial communities, where data acquisition is no longer the limiting factor for knowledge generation. Coupled with physical and biochemical techniques, this progress provides nano-resolution of food structure and microbial activity in the gut. A new frontier in current research that integrates information across fields alters and expands what is considered wholesome. Food, and the broader aspects of diet, are the primary modulator of the gut microbiota essential for homeostasis and prevention of chronic and infectious diseases. Understanding how food composition and structure alter the microbiota-host interaction is vital to engineering the next generation of foods that meet our nutritional needs while addressing safety and long-term well-being. This widening of scope bridges gaps between disciplines to improve the cross-feeding of ideas among experts interested in designing foods to be part of a diet that pushes the boundaries of current life expectancy. This Special Issue has collected studies from authors from five countries, providing insights into current research relating food ingredients with gut microbiota, whether healthy or not.

“Probiotics are live microorganisms that, when consumed in adequate amounts, confer a health benefit on the host” [1]. In order to advance the evidence for recommending their use, especially for preterm neonates, we need to know more about the microbial dynamics of the gut ecosystem over substantial periods of time. Wydau-Dematteis and colleagues [2], in this Special Issue, study the succession of bifidobacterial strains in preterm infants through their first 16 months and found that during the first year, the bifidobacterial population is unstable. These results are of central importance for developing probiotic combinations that are more likely to achieve a health benefit in preterm neonates, given the gut microbiota instability.

In the study by Fan et al. [3], the enterotype concept was extended from humans to a small herbivorous mammal, the plateau pika. When moved from the field into a controlled lab environment with a single feed source, the gut microbiota lost diversity, and one enterotype became dominant. Supplementing the lab diet with a plant secondary compound from the pika’s natural diet modulated the microbiota, increasing the fecal microbial diversity, emphasizing the role of diet in modulating the composition and potential activity of the gut microbiota. This knowledge will have an impact on our ability to preserve the native microbial diversity, maintaining resiliency in animal models of the gut microbiota. Moving from human and animal models to simulated gastrointestinal systems, the paper by Hong and colleagues (2021) [4] illustrates how probiotic supplementation of a stabilized gut microbiota with *Lactiplantibacillus plantarum* over a two-week period stimulated the production of butyrate and led to higher levels of riboflavin than the control treatment consisting of a non-riboflavin producing strain of the same species. The strain-specific activity underscores the importance of probiotic validation studies for ensuring the selection of strains with a higher probability of conferring a measurable health benefit. In vitro gastrointestinal models have an important role in screening dietary components’ effects on gut microbiota.

As another case in point for the vital contribution of in vitro models to screening processes, fecal slurries were employed by Vacca et al. [5] as a final step to narrow down the combinations of probiotic microbial strains, prebiotic oligosaccharides, and antioxidant plant extracts with the most promise for future testing in clinical studies as synbiotic formulations. Only 2 out of 25 strains passed all the preliminary tests, again emphasizing the significance of strain variability and selection. The final formulation showed the ability to modulate some metabolites (uremic toxin precursors, indole, *p*-cresol) in the fecal slurries from chronic kidney disease (CKD) patients, while few differences were present in fecal slurries from healthy subjects. These in vitro results highlight the challenges confronting the formulation of effective synbiotic food products, providing a measure of confidence moving forward into in vivo studies. 

Torres-Maravilla et al. [6] reviews evidence for dysbiosis associated with colorectal cancer (CRC), providing recommendations for biomarkers to diagnose CRC. The authors cover a wide range of preventive or therapeutic approaches, including probiotics, postbiotics, synbiotics, and next-generation probiotics as strategies to mitigate the onset and progression of this disease. Studies of the microbial species that are depleted in disease conditions have particular importance in defining new potential probiotic species and strains, which are now termed “next-generation probiotics” originating from healthy gut microbiota.

Overall, this Special Issue has brought together quite distinctive viewpoints that we can expect to cross-feed ideas between researchers working with in vitro models, formulation of food components, live animals, and humans towards the common goal of improving our diets. We hope you enjoy reading this selection of papers.
